# Shortening Leukocyte Telomere Length Associated With Elevated Blood Diabetes–Related Cardiovascular Risk Factor in Thai Adolescents

**DOI:** 10.1002/fsn3.70546

**Published:** 2025-08-05

**Authors:** Phennapha Luealai, Tippawan Pongcharoen, Nattira On‐Nom, Uthaiwan Suttisansanee, Piya Temviriyanukul, Wantanee Kriengsinyos, Chanakan Khemthong, Chaowanee Chupeerach

**Affiliations:** ^1^ Institute of Nutrition, Mahidol University Nakhon Pathom Thailand; ^2^ Nutrition Therapeutic Department Bangkok Hospital Headquarter Bangkok Thailand

**Keywords:** biochemical assessment, inflammatory marker, metabolic syndrome, teenager health

## Abstract

Leukocyte telomere length (LTL) is considered a reliable biological indicator of aging. LTL shortening has been associated with an increased risk of cardiometabolic markers among adults. However, evidence for an association in adolescents is limited. This cross‐sectional study examined the association between blood biomarkers of cardiovascular diseases and leukocyte telomere length in 59 Thai adolescents with mean age 15 years. Blood samples and anthropometric, clinical, and biochemical data were collected, with relative telomere length (RTL) measured using the quantitative polymerase chain reaction (qPCR) method. Results showed that RTL was negatively associated with Hemoglobin A1C (HbA1C) but positively associated with high density lipoprotein cholesterol (HDL‐C). Inflammatory Tumor necrosis factor alpha (TNF‐α) was positively correlated with RTL, with a strong association in female adolescents. After adjusting the confounding factors including age, gender, and body mass index (BMI) for age the multivariable models indicated that predictors of shortening RTL were higher levels of HbA1C (*β* = −0.444, *p* = 0.002) and lower in HDL‐C (*β* = 0.025, *p* = 0.002). The results suggested a positive association between telomere length and cardiometabolic risk factors such as HbA1C and HDL‐C in Thai adolescent subjects. To confirm these findings, a longitudinal study should be conducted in the future with a larger sample size with considering of environment factors.

## Graphical abstract

Association between leucocyte telomere length and cardiometabolic risk factor including HbA1C, HDL, and inflammatory cytokine; TNF‐ α in adolescent population.

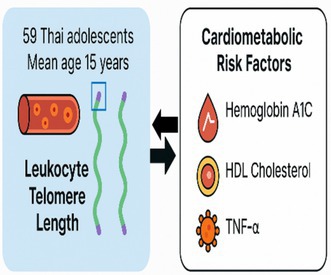

## 1 Introduction

The incidence of non‐communicable diseases (NCDs) among adolescents is a significant public health problem worldwide (Akseer et al. 2020). NCDs such as cardiovascular disease, diabetes mellitus, obesity, and cancer reduce the quality of life and increase mortality (Akseer et al. 2020). Children and adolescents are often exposed to harmful environmental factors and lifestyles such as tobacco and alcohol use, while sedentary lifestyles and unhealthy dietary habits increase stress. Nowadays, the risks of developing non‐communicable diseases (NCDs) from an early age are higher. Early detection of health risks and aging‐associated organ degeneration diseases can be improved using precision medicine‐guided diagnoses. Aging biomarkers including telomere length now offer the means to detect health risks at younger ages, especially in adolescents (Salameh et al. 2020).

An important biomarker for several diseases and longevity is telomere shortening. Telomeres are nucleoprotein structures containing TTAGGG tandem sequences (Blackburn 2000), together with a protein located at the tip of chromosomes that protects and preserves genome stability through several mechanisms (Chow et al. 2012; De Lange 2009). Telomeres become shorter after each mitotic division of somatic cells because of incomplete DNA replication and decline with age, resulting in cellular aging, cell death, or driving the cycle of somatic cells forward, which affect human health (Blackburn 2009; Factor‐Litvak et al. 2016; Gavia‐García et al. 2021). Evidence has shown that shorter leukocyte telomere length (LTL) is related to the risk of age‐degenerative diseases including cardiovascular disease (CVD), diabetes, obesity, hypertension, and dyslipidemia (Boniewska‐Bernacka et al. 2020; Kahrizi et al. 2022; Novau‐Ferré et al. 2023; Tellechea and Pirola 2017; Wang et al. 2016). Telomere shortening and increase in hydroxyl radicals may result from oxidative stress, inflammation, and aging (O'Donovan et al. 2011). Telomere length is not used in clinical practice but has shown potential as a biomarker for a range of chronic diseases.

A recent study determined telomere length as a possible biomarker of lifetime disease risk (Bosquet Enlow et al. 2018), while poor in growth has been associated with shorter telomeres in newborns and adolescents (Hjort et al. 2018; Sibert et al. 2021). A study of adolescent subjects (Gaydosh et al. 2020) found that relative telomere length (RLT) was associated with younger age and sociodemographic including higher household income. However, few studies have reported on the association between RLT and cardiometabolic risk factors including obesity in adolescents (Grunnet et al. 2019; Kliewer and Robins 2022) and type 2 diabetes mellitus (2DM) in young men (Grunnet et al. 2019). Adolescence is a critical period for the creation of lifestyle habits that can influence health outcomes later in life, and studying biomarkers in adolescents can help to identify early signs of risk factors for chronic diseases. Telomere length is associated with aging but few studies have focused on the relationship between leukocyte telomere length and blood biochemical parameters including cardiometabolic risk factors such as dyslipidemia, diabetes, or inflammatory state in adolescents. Therefore, this study clarified the association between leukocyte telomere length and blood cardiometabolic risk factors in Thai adolescents.

## 2 Materials and Methods

### 2.1 Study Design and Subjects

This cross‐sectional study consisted of 59 adolescent subjects with similar ages (mean age close to 15 years old) to avoid confounding in relative telomere length level. The data and blood samples were sourced from the “Association of early life exposure and long‐term health and cognitive development outcome in adolescents in Northeast Thailand” project, a community‐based study of adolescent nutrition and health conducted in Khon Kaen Province during 2013. The sample size calculation was in Supplement 1. The study was approved by the Research Ethics Committee of the Human Research Internal Review Board, Mahidol University (MU‐IRB), COA. No. MU‐CIRB 2022/062.1603. Informed written consent was obtained from all the study subjects.

### 2.2 Anthropometric and Clinical Assessments

Weight, height, and waist circumference were measured on the morning of data and blood collection. Weight was measured in kilograms using a digital scale (Seca digital scale model 813, Seca Corporation, Hamburg, Germany). Height in centimeters was measured using a measuring tape, with waist circumference measured in centimeters using an inelastic measuring tape. Weight and height data were used to calculate the body mass index (BMI) for age Z‐score to determine the nutritional status of the subjects following the WHO growth reference (2007) (Kulaga et al. 2010). Systolic and diastolic blood pressure (SBP and DBP, respectively) were obtained using an automatic blood pressure monitor. Each subject was asked to assume a sitting position before the measurements.

### 2.3 Blood Collection

The study participants were instructed not to eat or drink anything except water for at least 8 h before 10 mL of fasting blood collection at the study site (Khon Kaen Province). Serum was used for biochemical measurements including lipid profile, blood sugar levels, Hemoglobin A1C (HbA1C), and inflammatory markers. Ethylenediaminetetraacetic acid (EDTA)‐treated blood was kept at −80°C for further analysis of DNA.

### 2.4 Laboratory Analysis

Fasting blood sugar (FBS), serum triglycerides (TG), total cholesterol (TC), and high‐density lipoprotein cholesterol levels (HDL‐C) were analyzed by enzymatic assay. Low‐density lipoprotein cholesterol level (LDL‐C) was calculated using the Friedewald equation (Friedewald et al. 1972). Plasma insulin was measured by chemiluminescence immune assay, and HbA1C levels were measured by the high‐performance liquid chromatography (HPLC) technique. The inflammatory markers including interleukin‐6 (IL‐6) and tumor necrosis factor‐alpha (TNF‐α), were analyzed using the enzyme‐linked immunosorbent assay (ELISA) method following the suggested method in another study (Tuntipopipat et al. 2011). High‐sensitivity C‐reactive protein (hs‐CRP) was analyzed using the immunoturbidity method according to the laboratory procedure.

### 2.5 Telomere Length Analysis

#### 2.5.1 DNA Extraction

Genomic DNA was extracted from the peripheral leukocytes in EDTA‐treated blood using a Flexi Gene DNA Kit (Qiagen, Hilden, Germany). The extracted DNA concentration and purity were confirmed by a NanoDrop Lite Spectrophotometer (Thermo Scientific USA). DNA purity was determined by the ratio of absorbance values at 260 vs. 280 nm (normal range 1.8–2.0) and 260 vs. 230 nm (normal range 2.0–2.2).

#### 2.5.2 Relative Telomere Length Measurement

Relative telomere length (RTL) was analyzed by a quantitative polymerase chain reaction (qPCR) method, previously described in detail (Cawthon 2002), using a QuantStudio 5 Real‐Time PCR machine (Thermo Fisher, USA). In brief, this measures the average telomere length expressed as the ratio between telomere length (T) amplification products and single‐copy gene (S) products. Acidic ribosomal phosphoprotein PO (36B4) was used as the reference single‐copy gene for each DNA sample (O'Callaghan and Fenech 2011). Each 10 μL reaction consisted of a 20 ng DNA template, 1× PowerUp SYBR Green Master Mix, 100 nM telomere forward primer, and telomere reverse primer. The primer sequences were telomere forward (5′‐CGGTTTGTTTGGGTTGGGTTTGGGTTTGGGTTTGGGTT‐3′), and telomere reverse (5′GGCTGCCTTACCCTTACCCTTACCCTTACCCTTACCCT‐3′). The 36B4 primer concentrations were 250 nM of forward sequence (5′‐CAGCAAGTGGGAAGGTGTAATCC‐3′), and 36B4 reverse sequence (5′‐CCCATTCTATCATCAACGGGTACAA‐3′). The cycling conditions comprised an initial holding stage at 95°C for 10 min, followed by 40 cycles of 95°C for 15 s and 60°C for 1 min (Cawthon 2002). All samples were run in duplicate for quality control and checked for agreement between the duplicate values. Samples with high variable values (> 10%) were rerun and reanalyzed. Negative control and a melting curve were performed for telomere and 36B4 primers to check the efficiency and specificity of qPCR. The intra‐ and inter‐assay percentage coefficients of variation (%CV) were calculated based on the ratio of standard deviation and average mean values of the duplicates. The average intra‐assay %CV for telomere was 1.4% (range 0.04%–9.42%), while 36B4 was 0.8% (range 0.02%–9.08%). The inter‐assay %CV results of telomere and 36B4 were 1.3% and 0.5%, respectively. The T/S ratio for each sample was calculated using the formula:

$$\text{Calculated ∆Ct}=\mathrm{Ct}\;\left(\text{Telo}\right)-\mathrm{Ct}\left(36\text{B4}\right)$$

$$\text{Calculated}\;T/S\;\text{ratio}=2^{-\text{∆Ct}}$$

The relative T/S ratio was determined by dividing the T/S ratio of each sample by the mean T/S ratio of the entire adolescent subjects and expressed as relative telomere length (RTL) (Ventura Marra et al. 2019).

### 2.6 Statistical Analysis

Data were tested for normal distribution using the Kolmogorov–Smirnov test. Subject characteristics were analyzed using descriptive data and presented as the mean and standard error of the mean. The Student *t*‐test was used to examine the differences between the two gender‐independent groups, with Pearson's correlation test used to determine the relationship between the relative telomere length and all parameters. Simple linear regressions were used to investigate the possible association between each predictor and relative telomere length, while Enter linear regression analyses were used in full model and Backward design for final model. All statistical analyses were performed using SPSS Ver. 18 for Windows (Chicago, USA), with *p* < 0.05 considered statistically significant.

## 3 Results

### 3.1 Baseline Characteristics of the Subjects

Fifty‐nine subjects, 29 males and 30 females, were included in the analysis. The baseline characteristics of the participants, including anthropometric, clinical, and biochemical measurements, are presented in Table 1. No significant differences were observed in age, anthropometric, biochemical, and relative telomere length (RTL) measurements between males and females. Females tended to have a higher RTL than male subjects, but no significance was observed.

**TABLE 1:** Baseline anthropometric, clinical and biochemical data and nutritional status of subjects.

Variable Mean ± SE	Total *N* = 59	Male *N* = 29	Female *N* = 30
Age (years)	14.8 (0.06)	14.8 (0.06)	14.7 (0.05)
Waist circumference (cm)	68.02 ± 1.04	68.40 ± 1.26	67.66 ± 1.68
BMI for age (Z‐score)	−0.30 ± 0.15	−0.27 ± 0.21	−0.34 ± 0.22
Systolic blood pressure (mmHg)	105.94 ± 1.30	108.26 ± 2.02	103.71 ± 1.61
Diastolic blood pressure (mmHg)	61.65 ± 0.92	60.12 ± 1.32	63.13 ± 1.24
Nutritional status^a^			
Thin	6 (10.2)	3 (10.3)	3 (10.0)
Normal weight	45 (76.3)	23 (79.3)	22 (73.3)
Overweight	6 (10.2)	2 (6.9)	4 (13.3)
Obese	2 (3.4)	1 (3.4)	1 (3.3)
FBS (mg/dL)	91.75 ± 1.10	92.98 ± 1.26	90.56 ± 1.79
HbA1C (%)	5.62 ± 0.09	5.71 ± 0.16	5.53 ± 0.11
Insulin (μIU/mL)	2.14 ± 0.49	1.95 ± 0.84	2.33 ± 0.53
Triglyceride (mg/dL)	108.34 ± 6.45	106.07 ± 10.71	110.53 ± 7.49
Cholesterol (mg/dL)	137.20 ± 3.91	131.21 ± 4.32	143.00 ± 6.37
HDL‐C (mg/dL)	37.80 ± 1.73	38.07 ± 2.06	37.53 ± 2.79
LDL‐C (mg/dL)	75.14 ± 2.58	72.45 ± 2.90	77.73 ± 4.23
hs‐CRP (mg/L)	0.67 ± 0.16	0.97 ± 0.29	0.38 ± 0.10
IL‐6 (pg/mL)	311.74 ± 29.77	312.43 ± 42.37	311.05 ± 42.55
TNF‐α (pg/mL)	27.27 ± 4.59	22.37 ± 5.34	32.00 ± 7.39
Relative telomere length	1.00 ± 0.11	0.86 ± 0.09	1.13 ± 0.19

*Note:* Data are expressed as mean ± SE and ^a^as frequency. BMI for age (BAZ); thin = <−2SD, normal weight = −2SD to +1SD, overweight= > +1SD to <+2SD, obese = > +2SD. The Student *t*‐test showed differences in quantitative data between genders and ^a^ *Z*‐test for proportional data between genders.

Abbreviations: BMI, body mass index; FBS, fasting blood sugar; HbA1C, hemoglobin A1c; HDL‐C, high‐density lipoprotein cholesterol; hs‐CRP, high sensitivity C‐reactive protein; IL‐6, interleukin 6; LDL‐C, low‐density lipoprotein cholesterol; TNF‐α, tumor necrosis factor‐alpha.

### 3.2 Correlation Between Relative Telomere Length and Biochemical Parameters

**FIGURE 1:**
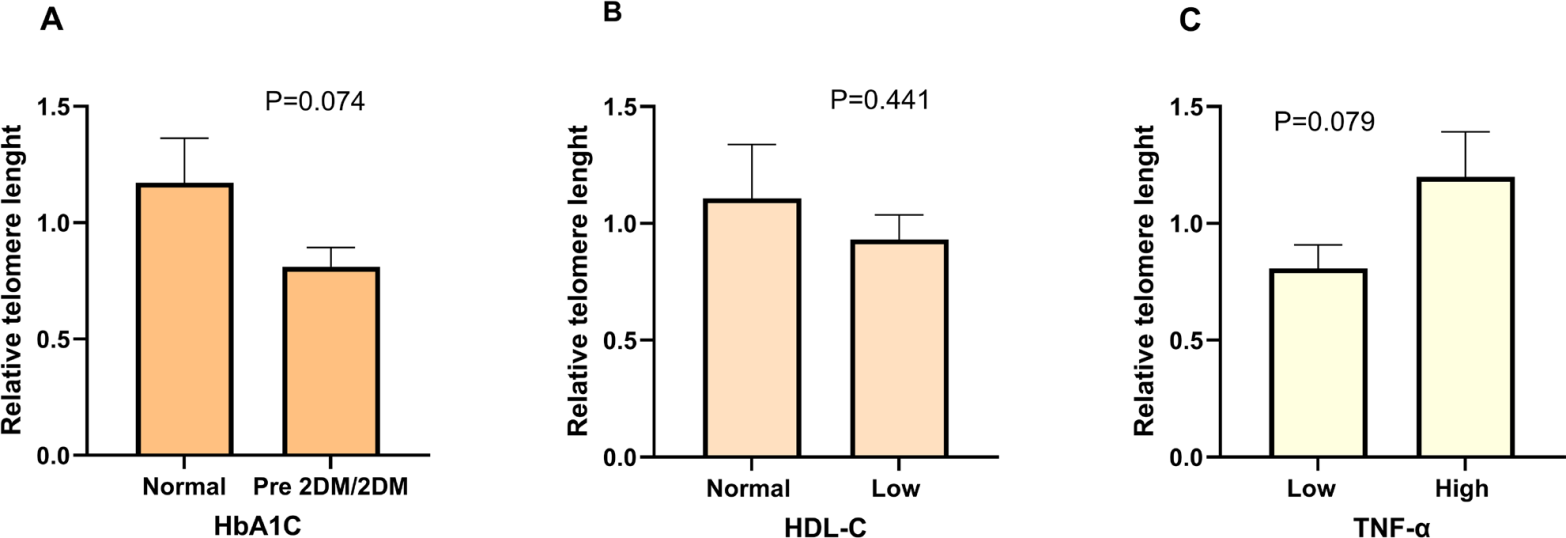
Relative telomere length between different classifications of HbA1C (A), HDL‐C (B), and TNF‐α (C). Data are presented as mean ± standard error of the mean (SE). DM: Type 2 diabetes mellitus. HbA1C: Normal < 5.7%, pre‐diabetes 5.7%–6.3%, diabetes ≥ 6.4%; HDL: Low < 40 mg/dL; TNF‐α: Low < 10.75 pg/mL (the median level). *p* value from the Student *t*‐test.

The bivariant correlation analysis between genders is presented in Table 2. In all adolescent subjects, relative telomere length (RTL) was significantly negatively correlated with HbA1C but positively correlated with HDL‐C levels. Insulin tended to be positively correlated with RTL, whereas blood sugar was negatively correlated. Different results were observed between genders. In male subjects, lower RTL significantly correlated with higher total cholesterol and LDL‐C but no correlation was found within the female group. The inflammatory markers hs‐CRP and IL‐6 were negatively correlated with RTL but with no statistical significance. Only TNF‐α gave a positive correlation with RLT in the female group. From Table 2, these results may be related among nutrition status such as 2DM, low HDL with telomere length and TNF alpha. Therefore, the stratified analysis by pre 2DM/2DM and normal group were compared (Figure 1). RTL tended to be shorter in subjects of 2DM groups by HbA1C cut‐off when compared with the normal group (*p* = 0.074). Similar to the subjects in low HDL‐C (< 40 mg/dL), RTL tended to be shorter than in normal HDL‐C levels. For TNF‐α, the low TNF group (less than median level; 10.75 pg/mL) tended to have a longer telomere than the higher RTL groups.

**TABLE 2:** Bivariate correlation of relative telomere length and anthropometric, clinical, and biochemical variables.

Parameter	All	Male	Female
BMI for age (Z‐score)	0.053	−0.036	−0.042
WC (cm)	0.011	0.115	0.032
SBP (mmHg)	−0.031	0.223	−0.032
DBP (mmHg)	0.056	0.205	−0.120
Fasting blood sugar (mg/dL)	−0.122	−0.119	−0.108
Insulin (μIU/mL)	0.142	0.408	0.013
HbA1C (%)	−0.286*	−0.177	−0.324
Triglyceride (mg/dL)	−0.008	−0.064	−0.070
Cholesterol (mg/dL)	−0.116	−0.384*	−0.082
HDL‐C (mg/dL)	0.260*	−0.071	0.351
LDL‐C (mg/dL)	−0.245	−0.370*	−0.246
hs‐CRP (mg/L)	−0.013	−0.020	−0.056
IL‐6 (pg/mL)	−0.147	−0.178	−0.053
TNF‐α (pg/mL)	0.113	−0.111	0.438*

*Note:* Pearson's correlation test was used to determine the correlation between the relative telomere length and anthropometry, clinical, and biochemical data.

* *p* < 0.05 was significant difference.

### 3.3 The Regression Analysis Between Telomere Length and Biochemical Parameters

To account for confounding factors in the analysis, multiple linear regression analysis was used to determine the association between relative telomere length and biochemical parameters, using RTL as a dependent variable (Table 3). After adjusting for age, gender, and BMI for age (Z‐score), higher HbA1C level and lower HDL‐C were shown to be a significant predictors of lower RTL (*b* = −0.320, 95% CI = −0.603; −0.037, *p* = 0.027 and *b* = 0.025, 95% CI = 0.010; 0.041, *p* = 0.002), and accounted for 24.4% (*R* ^2^ = 0.244) of the variance of relative telomere length. For the effect of TNF‐α, it disappeared (*p* > 0.05) after the adjustment.

**TABLE 3:** Multiple linear regression of relative telomere length determinants (*N* = 59).

Predictor	Full model			Final model		
	*β*	95% CI	*p*	*β*	95% CI	*p*
HbA1c	−0.320	[−0.603; −0.037]	0.027*	−0.444	[−0.714; −0.175]	0.002*
TNF α	0.005	[−0.002; 0.012]	0.144			
HDL‐C	0.020	[0.002; 0.038]	0.032*	0.025	[0.010; 0.041]	0.002*
Age	0.613	[−0.064; 1.289]	0.075			
Gender	0.220	[−0.195; 0.634]	0.292			
BMI for age (Z‐score)	−0.023	[−0.064; 1.289]	0.799			
Adjusted R^2^	0.157			0.244		

*Note:* Relative telomere length was a dependent variable. Adjusted variables included age, gender, and BMI for age. Enter linear regression analyses were used in full model and Backward design for final model. *β*; unstandardized coefficient, 95% CI; 95% confidence interval.

* Significant with *p*‐value < 0.05.

## 4 Discussion

This study investigated the association of relative leukocyte telomere length (RTL) and blood cardiometabolic biomarkers among Thai adolescents. In this study, relative telomere length (RTL) negatively correlated with HbA1c, with links to type 2 diabetes and positive correlated with HDL‐C with link to a risk of CVD development. As in previous studies, negative associations were found between RTL, FBS, and HbA1C levels in adolescents (Gaydosh et al. 2020) and young healthy men (Grunnet et al. 2019). Subjects with altered glucose levels had shorter telomere lengths than those with normal glucose levels (Guo et al. 2011; Todendi et al. 2020; Zhao et al. 2014).

Associations between leukocyte telomere length and lipid profiles were also found. Leukocyte telomere shortening was associated with low levels of HDL‐C, a type of cholesterol that helps to remove excess cholesterol from the body and protects against cardiovascular disease. Similar to our results, the Bogalusa Heart Study also found a positive relationship between leukocyte relative telomere length (RTL) and high‐density lipoprotein cholesterol (HDL‐C) during childhood, with low HDL‐C levels being one of the significant factors influencing telomere attrition (Chen et al. 2009; Huzen et al. 2014). HDL‐C has anti‐inflammatory and antioxidant properties that may help to reduce age‐related telomere shortening. HDL‐C also promotes telomerase activation, an enzyme that helps to maintain telomere length, resulting in decelerated telomere shortening in this population (Chen et al. 2009; Khera et al. 2011). However, there was no association between triglyceride level and RTL in the present study. This observation aligns with several previous reports that have similarly yielded inconclusive or mixed findings, suggesting that the relationship between triglyceride levels and RTL remains unclear (Hu et al. 2022; Révész et al. 2015).

In this study, shorter RTL was associated with elevated total cholesterol, triglyceride, and LDL levels in all subjects, but no statistical significance was observed. Similarly, a recent study on Brazilian children and adolescents found a trend of shorter RTL with three to four lipid profile alterations (Todendi et al. 2020). The author suggested that elevated total cholesterol levels might stimulate cell death and increase the synthesis of reactive oxygen species (ROS). Moreover, higher LDL‐C levels might reduce serum total antioxidant status (TAS), with both mechanisms contributing to an accelerated telomere‐shortening process (Ludwig et al. 1982; Weng et al. 2019). However, only male adolescents showed a negative correlation between RTL and lipid profiles including total cholesterol and LDL‐C. This may reflect sex‐specific differences in telomere biology and lipid metabolism, which require further investigation.

The association between RTL and inflammatory markers has been investigated by several previous studies. In this study, a positive correlation between RTL and TNF‐α was observed in female adolescents. Moreover, females had longer telomeres than male adolescents (Lansdorp 2022). These might be the effect of hormonal modulation on inflammatory cytokines that could relate to telomere length. Adolescent girls change the body composition that could increase in fat mass, especially central adiposity, which is related to higher TNF‐α levels due to adipose tissue‐derived cytokine secretion (Nemet et al. 2003). Higher levels of TNF‐α were associated with longer telomere length, correlating with a previous study, which concluded that leukocyte telomere length was positively related to the pro‐inflammatory cytokine hs‐CRP (Flannagan et al. 2020). However, no association of hs‐CRP and telomere was found in the present study. For the mechanism among telomere and cytokines, TNF‐α and IL‐6 promote telomerase activity through the nuclear factor kappa‐light‐chain‐enhancer of activated B cells (NF‐κB) by promoting the transcription of the telomerase reverse transcriptase gene, which encodes the catalytic subunit of telomerase, leading to increased telomere length (Chung et al. 2017; Gizard et al. 2011; Jose et al. 2017; Wang and Bennett 2010). However, TNF‐α is a central inflammatory cytokine whose expression and activity are influenced by multiple factors, including exposure to air pollution or promoter methylation. Recent evidence suggests that TNF‐α methylation may mediate the biological effects of environmental stressors, potentially influencing both cytokine levels and downstream consequences, such as telomere attrition (Dolcini et al. 2024). No association of IL‐6 was also observed in the present study, which needs further study to clarify the association of telomere and inflammatory markers among the adolescent population.

This study had several strengths. By using the same age in adolescent subjects, we avoided the effect of older age on telomere length. Confounding factors were adjusted in multiple regression to analyze for HbA1C and HDL‐C as predictors for relative telomere length. The adolescence in this study have no serious illness therefore these data could be used as a biomarker in general pediatric population. By contrast, there were also limitations. First, the cross‐sectional design restricted the potential for causative relationships, second, the number of subjects should be increased to clarify some non‐significant results, third, the lifestyle factors including diet, sleep, physical activity, air pollutant need to be combined for the telomere length analysis in future study.

## 5 Conclusions

This study observed that biomarkers of cardiovascular disease including HbA1C and HDL were associated with leukocyte telomere length (RTL) in Thai adolescents, concurring with previous research on adults. The results contribute to the existing body of knowledge on the relationship between telomere length and cardiometabolic risk factors in younger age groups. These findings may serve as a basis for generating hypotheses in future longitudinal or interventional studies aimed at understanding biological aging and the early development of age‐related diseases.

## Author Contributions

**Phennapha Luealai:** conceptualization (supporting), formal analysis (equal), investigation (equal), methodology (supporting), resources (supporting), software (supporting), writing – original draft (equal), writing – review and editing (equal). **Tippawan Pongcharoen:** conceptualization (equal), data curation (equal), formal analysis (equal), funding acquisition (supporting), investigation (equal), methodology (equal), project administration (equal), resources (equal), software (equal), supervision (supporting), validation (equal), visualization (supporting), writing – original draft (equal), writing – review and editing (equal). **Nattira On‐Nom:** conceptualization (equal), data curation (equal), formal analysis (equal), investigation (supporting), methodology (equal), project administration (equal), resources (equal), software (equal), supervision (equal), validation (equal), visualization (equal), writing – original draft (equal), writing – review and editing (equal). **Uthaiwan Suttisansanee:** conceptualization (supporting), data curation (supporting), investigation (supporting), methodology (supporting), resources (supporting), software (supporting), validation (supporting), visualization (supporting), writing – original draft (supporting), writing – review and editing (supporting). **Piya Temviriyanukul:** data curation (supporting), formal analysis (supporting), investigation (supporting), methodology (supporting), resources (supporting), software (equal), supervision (supporting), validation (supporting), visualization (supporting), writing – original draft (supporting), writing – review and editing (supporting). **Wantanee Kriengsinyos:** data curation (supporting), validation (supporting). **Chanakan Khemthong:** investigation (supporting). **Chaowanee Chupeerach:** conceptualization (lead), data curation (lead), formal analysis (lead), funding acquisition (lead), investigation (equal), methodology (lead), project administration (lead), resources (lead), software (lead), supervision (lead), validation (lead), visualization (lead), writing – original draft (lead), writing – review and editing (lead).

## Ethics Statement

The study was conducted in accordance with the Declaration of Helsinki, and approved by Mahidol University Central Institute Review Board (COA. No. MU‐CIRB 2022/062.1603, date of approval: 17 May 2022).

## Consent

Informed consent was obtained from all subjects involved in the study. Written informed consent has been obtained from the patient.

## Conflicts of Interest

The authors declare no conflicts of interest.

## Data Availability

The authors have nothing to report.
